# Involvement of the *Gli3 (Extra-Toes)* Gene Region in Body Weight in Mice

**DOI:** 10.1100/tsw.2007.40

**Published:** 2007-01-26

**Authors:** Benoît Martin, Eve Lapouble, Yohan Chaix

**Affiliations:** ^1^ Laboratoire de Neurobiologie, Université d'Orléans, B.P. 6759, 45067 Orléans Cedex 2, France; ^2^ INRA, UMR1079 - Systèmes d'Elevage Nutrition Animale et Humaine, 35590 Saint Gilles, France

**Keywords:** development, obesity, QTL mapping, mouse chromosome 13, extra-toes gene, body weight, gene mutation

## Abstract

The mutation extra-toes (Gli3^Xt-J^) on chromosome (Chr) 13 of the mouse is known to be involved in the development of the skeleton. The only visible manifestation is the presence of an extra digit on each hind foot. Here we report evidence from several experiments that Gli3^XtJ/+^ mice weigh more than littermate Gli3^+/+^ mice, suggesting an effect on body weight of *Gli3* or of a gene tightly linked to it on Chr 13. Four independent experiments in different environments were conducted on mice with different genetic backgrounds derived from the C3XtEso Gli3^Xt-J/+^ E^so/+^ linkage testing strain and the JE/Le strain at adult age. The analyses have shown an association between the Gli3^Xt-J^ allele and a body weight increase of about 6.5%. This effect is genetically dominant. It would appear that if the gene of interest is not *Gli3* itself, it must be very close to this locus. Indeed, the expected size for this fragment is 7.9 ± 5.3 cM. The manifestation of this gene, observed in two animal facilities and on different genetic backgrounds, is consistent with the idea that the effect of the gene(s) is displayed in a stable manner. It accounts for a variation of 6.5% of body weight.

## INTRODUCTION

*Extra-toes (Xt)* is a mutation of the *Gli3* gene (*GLI-Kruppel family member GLI3*) with semi-dominant effect involving the skeleton on mouse chromosome (Chr) 13[1,2]. Its effects are only detected in animals heterozygotic for the mutation from the presence of extra digits on the preaxial side of fore and hind feet. Heterozygous mice are fully viable and breed freely with a normal behavior during their life, but homozygous mice die *in utero* or at birth due to a wide range of abnormalities. These include edema, paddle-shaped feet with up to nine digits, hemimelia, and gross malformations of the brain, central nervous system, and sense organs. *Xt*, the first mutant allele to be found, arose spontaneously in the control series of a radiation experiment at the MRC Radiobiological Research Unit at Harwell[3,4] and multiple allelic forms have been found since[5]. See also http://www.informatics.jax.org/javawi2/servlet/WIFetch?page=markerDetail&key=9121.

Gli3$^{Xt-J}$ (*extra-toes Jackson*) appeared in C3H/HeJ mice at The Jackson Laboratory (Bar Harbor, ME) and was subsequently outcrossed in the production of the STX/Le inbred linkage testing strain. At generation of inbreeding 71 (F71), production of a C3HeB/FeJ congenic inbred strain bearing the hybrid genotype Gli3$^{Xt\text{-}J/+}$ and another mutation named E$^{so}$ (sombre - Chr 8) by repeated backcrosses was begun[6]. In 1989, we received this line, C3HeB/FeJ-SX Gli3$^{Xt\text{-}J/+}$ E$^{so/+}$, at three generations of backcrosses (N3) from The Jackson Laboratory. A substrain, C3XtEso Gli3$^{Xt\text{-}J/+}$ E$^{so/+}$, has been derived from this line and has been maintained in our laboratory by forced heterozygosis at both the *Gli3* and *e* loci since this time (despite the inbreeding protocol, one or several genes are voluntarily maintained heterozygous by crossing hererozygous with homozygous breeders for this or these gene(s)). Presently, the mouse (sub)strain is at generation N3F30 (N*x*F*y* means *x* backcrosses followed by *y* brother-sister crosses with forced heterozygosis at one locus or several loci).

It should be noted that the forced heterozygosis breeding protocol results not only in maintaining *Xt* at the heterozygous level, but also a chromosomal segment surrounding the *Xt* gene. Thus, when performing within-strain studies for a trait of interest, by comparing a population carrying a segment with a marker in the homozygous (wild-type) form with the population carrying this same segment with the marker in the heterozygous form, any observed difference may be due to one or more unfixed loci on this chromosomal segment, with the marker itself being one candidate among all the other genes included in the fragment.

The genetic background of our strain was not, of course, inbred at N3F1 and was varying over the generations of brother-sister matings. Moreover, this strain has been maintained in two different breeding labs between generation N3F1–7 and N3F25. Here we report evidence from several experiments that Gli3$^{Xt\text{-}J/+}$ mice weigh more than littermate Gli3$^{+/+}$ mice, whatever the environment and genetic background. This suggests a stable effect on body weight of *Gli3* or of a gene tightly linked to it on mouse Chr 13.

## MATERIALS AND METHODS

Experiments were conducted at the Université Paris V - UFR Biomédicale des Saint-Pères for the generations N3F1 to N3F7 and at the Université d'Orléans for the generation N3F25. Animals were reared under standard conditions of: 24°± 1°C, a 12:12-h photoperiod with lights on at 8:00 a.m., water and Souriffarat (IM UAR) food available *ad libitum*, and dust-free sawdust bedding at both universities. The environmental differences at the two breeding facilities were not voluntarily different, but small differences were noted, such as painting color, bulb lighting, organization of the cages, etc. Litters having fewer than four pups were discarded and the others culled to six pups. Supernumerary pups were selected at random at day 1 or 2 and sacrificed. Animals were weaned at 30 ± 2 days of age and housed with two to 11 like-sex littermates in plastic boxes measuring 36 × 16 × 17 cm, 36 × 22 × 17 cm, and 36 × 38 × 18 cm for groups of subjects of 2–6, 7–9, and 10–12, respectively.

At generations N3F1 to N3F7, we crossed C3XtEso Gli3$^{Xt\text{-}J/+}$ mice with JE/Le inbred mice. Subsequently, C3JEF1 Gli3$^{Xt\text{-}J/+}$ and C3JEF1 Gli3$^{+/+}$ females were crossed with JE/Le males to obtain the backcross populations (C3JEF1)JE Gli3$^{Xt\text{-}J/+}$ and (C3JEF1)JE Gli3$^{+/+}$. All mice were weighed on the same day of age with a maximal variation of one day.

A three-way ANOVA with sex and genotype at the *Gli3* and *e* loci as independent main factors was performed for each of the four test populations.

## RESULTS

The C3XtEso mice born in our colony were systematically weighed at the age of 77 ± 6 days during two periods of time, at generations N3F1–7 and N3F25. In addition, we generated two other populations, C3JEF1 and C3JEN2, obtained from C3XtEso and an intercross or a backcross with JE/Le, another inbred strain. Both these populations were also bred and weighed in each of the generations from N3F1 to N3F7. The data are presented in Table 1.

**TABLE 1**
**Body Weight and Age (Mean ± SEM) and Sample Sizes for Each Population***

| Mice | Sex | Genotype for *Gli3* | | | | Body Weight Increase |
|---|---|---|---|---|---|---|
| | | +/+ | | *Xt-J*/+ | | |
| | | N | Weight (g) ± SEM | N | Weight (g) ± SEM | |
| C3XtEso (N3F1-7) | F | 134 | 22.27 ± 0.16 | 117 | 22.84 ± 0.19 | 2.56% |
| C3XtEso (N3F1-7) | M | 143 | 27.38 ± 0.20 | 139 | 29.66 ± 0.21 | 8.33% |
| C3XtEso (N3F25) | F | 30 | 20.06 ± 0.29 | 25 | 20.91 ± 0.43 | 4.24% |
| C3XtEso (N3F25) | M | 35 | 23.67 ± 0.48 | 31 | 25.81 ± 0.41 | 9.04% |
| C3JEF1 | F | 50 | 22.38 ± 0.23 | 66 | 23.91 ± 0.25 | 6.84% |
| C3JEF1 | M | 40 | 29.75 ± 0.42 | 51 | 31.18 ± 0.41 | 4.81% |
| (C3JEF1)JE | F | 148 | 18.88 ± 0.23 | 50 | 20.48 ± 0.34 | 8.47% |
| (C3JEF1)JE | M | 156 | 24.28 ± 0.26 | 51 | 26.45 ± 0.49 | 8.94% |

* Since no $E^{so}$ effect has been revealed with the analysis, populations were pooled for $E^{so}$ and, for the sake of simplicity, only *Gli3* genotypes are mentioned.

ANOVA shows an absence of effect on body weight for the Eso gene, but systematic sex ($p < 0.0001$ for all comparisons) and Gli3 effects. For the Gli3 factor, we have F = 55.00 ($p < 0.0001$), F = 12.49 ($p < 0.0006$), F = 20.10 ($p < 0.0001$), and F = 29.17 ($p < 0.0001$) for the respective populations C3XtEso (N3F1–7), C3XtEso (N3F25), C3JEF1, and C3JEN2. No significant interaction between *Gli3* and sex was found except for C3XtEso (N3F1–7) mice.

## DISCUSSION

The $\text{Gli3}^{Xt\text{-}J}$ genotype is associated with an increase of the body weight by about 10% compared to the wild-type $\text{Gli3}^{+}$. This suggests that the *Gli3* gene or gene(s) strongly linked to *Gli3* play a role in body weight. This increase was found systematically within the C3XtEso strain itself at two points during the inbreeding process, but also when this strain was intercrossed or backcrossed with a different strain. Its effect is not affected, or is affected in a very limited manner, by environmental factors since the two experiments run on C3XtEso at N3F1–7 and N3F25 were conducted at two different locations with a 10-year interval. Table 1 shows that, at equivalent ages, the four populations C3XtEso (2 genotypes *Gli3* × 2 sexes) show an overall decrease of about 11% of their body weight between generations N3F1–7 and N3F25. This reduction can be explained either by a genetic fixation of other genes during the inbreeding process and/or by environmental effects (including interactions between both). The preservation of the *Gli3* effect throughout the generations despite this global reduction of the body weight suggests that the effect of the *Xt* gene is relatively independent of the general genetic background. Moreover, the significance observed in the within-strain comparison for the C3JE F1 population suggests that $\text{Gli3}^{Xt\text{-}J}$ has a dominant effect on body weight. Taken together, these data suggest a notable, stable, and dominant effect of $\text{Gli3}^{Xt\text{-}J}$ genotype on body weight.

A slight increase in body weight of newborn for the original mutation of *Gli3* compared to wild type has been reported[1]. However, in the same report, heterozygotes, apparently at older ages, were found to be the same size and body weight as normal littermates. Two hypotheses are suggested by these results: (1) *Gli3* mutations cause an increase in body weight and the effect on body weight of the various *Gli3* alleles is allele-specific and (2) a locus linked to *Gli3* is responsible for the effect on body weight in our experiment. These effects would, therefore, be independent of the one previously reported by Johnson[1].

Several experiments have shown an involvement of proximal Chr 13 in body weight[7,8,9,10]. As far as we investigated, none of them identified a candidate gene, but all are compatible with our result, especially *Bdw1* and *Bdw15,* which have been positioned at 9 and 10 cM, respectively. Effectively, the size of the intact heterozygous segment surrounding a gene maintained with forced heterozygosis depends directly on the number of generations and on the length of the chromosome and the position of the gene on the chromosome[11,12,13]. The average size of the heterozygous segment decreases dramatically over generations. Therefore, given that the increase in body weight was also observed in the N3F25, it would appear that if the gene of interest is not *Gli3* itself, it must be very close to this locus. Of course, we cannot reject an oligogenetic effect where the supposed gene would be in fact several genes. Indeed, according to Naveira's equation[13], the expected size for this fragment is 7.9 ± 5.3 cM. A future PCR investigation should make the heterozygous segment specific and define whether the candidate genes previously located in the proximal Chr 13 remain candidates considering the new location and size.

## ACKNOWLEDGMENTS

The preparation of this article was supported by the Centre National de la Recherche Scientifique (CNRS), the Université Paris V - René Descartes, UFR Biomédicale des Saints-Pères, and the University of Orléans. We are indebted to Zohra Sérégaza (Aix-en-Provence, France) for her help in the experiments and Dr. Paul Neumann (Dalhousie University, Halifax - Canada) for critical suggestions on the manuscript.
